# The effects of episodic simulation on the cognitive and emotional expectancies related to personal goals

**DOI:** 10.1007/s00426-026-02369-4

**Published:** 2026-09-15

**Authors:** Rachel J. Anderson, J. Helgi Clayton McClure, Jennifer Shevchenko, Kevin J. Riggs, Stephen A. Dewhurst

**Affiliations:** 1 School of Psychology and Social Work, University of Hull, Cottingham Road, Hull, HU6 7RX UK; 2 School of Education, Language and Psychology, York St John University, York, UK

## Abstract

Episodic simulation influences future expectancies such as likelihood, controllability, and anticipated emotions of future events. We extended the use of episodic simulation to expectancies associated with personal goals. Participants listed personal goals and provided ratings of cognitive and emotional expectancies. They then completed one of four tasks (goal-related episodic simulation, goal-unrelated episodic simulation, neutral imagery, or letter-search) before providing two further sets of ratings. We also measured depressive symptoms. In line with hypotheses, results showed that ratings of controllability increased following both episodic simulation tasks for those experiencing high levels of depressive symptoms. This was maintained after a short 10-minute delay for those who had completed the goal-unrelated episodic simulation. These findings are in line with the notion that the effects of positive episodic simulation generalise to expectancies about future events beyond those that form part of the training itself, by fostering a more optimistic orientation. Findings for other expectancy ratings were not in line with hypotheses, with no beneficial changes in likelihood, importance, or anticipated disappointment. Potential reasons for the inconsistencies with previous literature are discussed, as are the implications for future work exploring the effects of episodic simulation on goal-oriented motivation and achievement.

Personal goals represent our aims, dreams and ambitions that are realistically obtainable. They often require a degree of effort to achieve, but this effort is offset by the anticipated reward that accompanies goal success (MacLeod, 2017). Having set personal goals, people develop mental representations that serve to guide their pursuit. These include cognitive and emotional expectancies about the goal. For example, they will develop expectancies about the likelihood of goal success and its perceived controllability, as well as beliefs about how they will feel upon achieving the goal (Dweck, 2017). Moreover, these expectancies have an effect on goal-oriented behaviour: someone is unlikely to be motivated and to expend effort in working towards a goal that they do not foresee as pleasurable, think is unlikely to be achieved, or is outside of their control (Baumgartner et al., 2008; Carver & Scheier, 1998; Clayton McClure & Cole, 2022). It seems likely then that cognitive and emotional expectancies of personal goal success are a key influence on goal-related motivation and behaviour. A question arises, therefore, as to how these cognitive and emotional expectancies might be modified, with previous research suggesting that techniques incorporating mental imagery may serve as a useful tool in this regard.

Mental imagery refers to the generation of mental representations in the absence of external stimuli. Mental images function like a weak form of perception with sensory information activated as if the situation is experienced in the here and now (Pearson et al., 2015). One type of future-oriented mental imagery, termed episodic simulation, has garnered particular interest as a potential ‘motivation amplifier’ (Ji et al., 2021; Renner et al., 2019). Defined as the construction of detailed mental representations of specific future events relevant to the self (Schacter et al., 2017), episodic simulation has been argued to underpin other forms of prospective thinking such as the expectancies one makes about the future (Szpunar et al., 2014). For instance, Kahneman and Tversky’s (1982) simulation heuristic suggests that judgements about the likelihood of an event are often based on how easily the event can be mentally envisaged. Schacter et al. (2008) have argued that episodic simulation provides an emotional foretaste of the event upon which one makes judgements about the event’s probable emotional consequences.

It seems likely, therefore, that modifying someone’s episodic simulation abilities would impact their expectancies about personal events in their future. Thus, training in episodic simulation could be a useful tool within interventions targeted at improving future-oriented thinking, with the aim of improving motivation and behavioural engagement. A growing body of evidence supports this idea. Experimental studies have demonstrated that brief inductions of repeated episodic simulation increase the episodic detail and vividness of future events (Boland et al., 2018; Hallford et al., 2022a); make future episodes feel more plausible, controllable and likely to occur (Boland et al., 2018; Hallford et al., 2022b; Szpunar & Schacter, 2013); and increase the anticipated and anticipatory pleasure associated with future events (Hallford, Farrell et al., 2022; Ji et al., 2021; Renner et al., 2019).

It has yet to be established, however, whether training in episodic simulation can be used to modify the expectancies we have about our own personal goals. The aforementioned studies asked participants to make predictions about possible future events. While some of these studies asked participants to generate events that would be positive or rewarding (e.g. Hallford, Farrell et al., 2022; Ji et al., 2021; Renner et al., 2019), they did not explicitly ask participants to generate and make predictions about their own personal goals. This is important if the aim is to facilitate goal attainment by modifying the expectancies an individual holds about these goals. Therefore, the current investigation explored the impact of brief positive simulation training (PST) on cognitive and emotional expectancies related to personal goals.

At the outset of the study participants listed their personal goals and, for their most important goals, provided a series of ratings to index cognitive expectancies (likelihood, controllability, importance) and emotional expectancies (anticipated happiness accompanying goal success, anticipated disappointment accompanying goal failure). They were then assigned to one of four experimental manipulations: goal-related PST; goal-unrelated PST; neutral imagery task; or a letter search control task. The control task required participants to engage in visual search and monitoring but not the use of visual imagery. Subsequently, participants rated goal expectancies a further two times, once immediately after the experimental manipulation and again after completion of a 10-minute filler task. This design allowed us to test whether it is goal-related episodic simulation per se, rather than general (goal-unrelated) positive episodic simulation or even generic imagery engagement that affects goal-related expectancies. It also allowed us to establish whether any beneficial effects of PST were transitory or whether they remained after a short ten-minute delay.

How might PST affect these cognitive and emotional expectancies? There are two possibilities identified in the literature. One group of researchers argue that episodic simulation functions as a ‘motivational amplifier’ because it allows a person to mentally pre-experience the future event which, in turn, influences the cognitive and emotional expectancies they hold about that event (e.g., Ji et al., 2021; Renner et al., 2019). On this view then we might predict that episodic simulation needs to be goal-related to impact one’s expectancies about those goals. A second group of researchers, however, report findings that suggest the effects of episodic simulation training can generalise to expectancies about future events beyond those that form part of the training itself (e.g., Anderson et al., 2024; Boland et al., 2018; Hallford et al., 2023). These studies suggest that the repeated mental pre-experiencing of positive events can modify an individual’s optimistic orientation such that, in general, they see all positive events, *including their personal goals*, as more likely to occur and be pleasurable. If this is the case, then goal-related and goal-unrelated PST should have similar effects on future expectancies. Despite their differences, both of these proposals suggest that it is the *mental pre-experiencing* of a positive future that impacts future expectancies. Research investigating simulation as a ‘motivational amplifier’ is focused on rewarding events, which are inherently positive (e.g., Ji et al., 2021; Renner et al., 2019). Meanwhile, studies that directly compared the simulation of positive events with neutral simulation tasks found that the effect is confined to positive simulation tasks (e.g., Anderson et al., 2024; Boland et al., 2018). Together, this suggests that generic mental imagery, that is neither positive nor episodic (e.g. neutral scene construction), would not confer the same benefit as the PST tasks (goal-related and goal-unrelated). Furthermore, comparison of the two PST tasks could provide insight into the mechanism underlying the benefits. 

Training in episodic simulation as a method of improving goal-related expectancies is pertinent within the context of depression. Elevated levels of depressive symptomatology are associated with difficulties in motivation and goal pursuit (Anderson et al., 2023; Dickson et al., 2016, 2017). These difficulties with goal pursuit seem to be underpinned by a pessimistic predictive style whereby depressive symptomatology is associated with pessimistic predictions about goal success, with lower perceived control over, and expectancies of, goal success along with muted positive emotions (e.g. joy, pleasure) anticipated to accompany goal achievement (Anderson et al., 2023; Dickson et al., 2011; Gamble et al., 2021). Elevated levels of depressive symptomatology are also associated with difficulties in episodic simulation; moreover, it seems that *positive* episodic simulation is particularly affected, with depressed individuals having difficulty generating vivid simulations of positive, but not negative, future occurrences (e.g., Bjärehed et al., 2010; Holmes et al., 2008; MacLeod et al., 1997; MacLeod & Byrne, 1996; MacLeod & Salaminiou, 2001; Morina et al., 2011; Stöber, 2000). Therefore, these episodic simulation difficulties may sit at the heart of a pessimistic predictive style which feeds into the goal-related motivational difficulties evident in those experiencing elevated levels of depression. If positive episodic simulation is to be useful as a tool for therapeutic intervention, it is important to establish if it is effective in individuals with a higher level of depressive symptomatology. Therefore, a further aim of the current study was to investigate whether any effects of PST differed as a function of depressive symptomatology. For this purpose, we treated severity of depressive symptoms as a continuous variable to reflect the evidence that they exist along a continuum of severity throughout the population (e.g., Rodríguez et al., 2012).

To summarise, previous theory and research suggest that engaging in positive episodic simulation should lead to improvements in expectancies about personal goals (e.g. more likely to be achieved, more controllable etc.). Therefore, we hypothesised that participants who undertook brief inductions in PST, either goal-related or goal-unrelated, would report significantly higher post-manipulation goal expectancy ratings compared to those who completed the neutral imagery and letter search control tasks. If the relative improvements were only evident following the goal-related PST task, then this would lend support to the notion that positive episodic simulation acts as a ‘motivational amplifier’. Alternatively, if the effects were present for both PST tasks this would lend support to the proposition that positive episodic simulation elicits a more optimistic orientation. Finally, in line with previous research demonstrating that the beneficial effects of positive simulation extend to individuals experiencing high levels of depressive symptomatology (e.g., Anderson et al., 2024; Boland et al., 2018; Hallford et al., 2023), it was hypothesised that any effects within the current study would be evident across individuals irrespective of their current depressive symptom level.

## Method

### Participants

Ethical approval was granted by the authors’ institutional ethics committees, with procedures used in this study adhering to the tenets of the Declaration of Helsinki. 189 undergraduate and postgraduate students at the authors’ institutions participated in the study, either for research participation credits or a £12 Amazon e-voucher. Two participants were excluded due to procedural errors leading to missing data. The final sample (*N* = 187) comprised 111 females, 70 males and 6 individuals reporting other gender identities or declining to report their gender. Participant ages ranged from 18 to 59 years (M = 26.13, SD = 8.66). CESD-R depressive symptom scores ranged from 0 to 56 (M = 14.04, SD = 12.09). No a priori power analysis was conducted, but calculations in G*Power (Faul et al., 2007) suggest that *N* = 187 would be sufficient to detect medium between-group effects of η_p_^2^/*f*^2^ = 0.06 to 0.08 in aggregate ANOVA/regression (power = 0.80−0.95). The use of multilevel linear models means that actual power is likely to be higher in the main analyses.

Participants were randomly allocated to one of four experimental conditions: related-PST (*n* = 46), unrelated-PST (*n* = 47), neutral imagery control (*n* = 48) or letter search control (*n* = 46). Demographic information (age, gender details, CESD-R score) as a function of the four conditions can be found in Table 1.

**Table 1:** Age, Gender and CESD-R Scores a Function of Experimental Condition

	Condition			
	Related-PST	Unrelated-PST	Neutral Imagery	Letter Search
Age (Mean, SD)	25.09 (8.54)	27.00 (8.22)	28.33 (9.26)	23.98 (8.18)
Gender (M:F:Other)	12:32:2	15:31:1	27:20:1	16:28:2
CESD-R Score (Mean, SD)	14.50 (10.86)	16.36 (14.41)	10.35 (9.07)	15.07 (12.93)

Chi square analysis established that this random allocation to experimental condition did not lead to differences in gender frequencies across groups, *X*^*2*^(6, *N* = 187) = 11.03, *p* = .09. Furthermore, one-way between-subjects ANOVAs established that the four conditions did not differ significantly in age, *F*(3,183) = 2.42, *p* = .07, or CESD-R scores, *F*(3,183) = 2.24, *p* = .09.

### Materials

#### Centre for epidemiologic studies depression scale - revised (CESD-R)

The CESD-R (Eaton et al., 2004) is a 20-item self-report inventory measuring depressive symptomatology in the general population, as defined by the Diagnostic and Statistical Manual of Mental Disorders (DSM-5; American Psychiatric Association, 2013). Participants rate frequency of symptoms using 5-point Likert scales ranging from ‘Not at all or less than 1 day’ (0) to ‘Nearly every day for 2 weeks’ (4). A total score of 0–80 is then produced by summing scores for all items. The CESD-R has demonstrated strong psychometric properties in adult community samples (Van Dam & Earleywine, 2011).

#### Goal production task

This task required participants to write down as many goals as possible for each of three different future time periods in ascending order (next week, next year, next 5–10 years). Three minutes were given per time period. Goals were defined as ‘things you would like to achieve in the future’. After the ‘next 5–10 years’ time limit elapsed, participants numbered (1–3) their top three *most important* goals from each time period. In the related-PST condition only, participants also generated an achievement event for each of these nine goals. This was defined as a specific episode that would symbolize achievement of each goal. For example, for the goal ‘graduate’, the achievement event could be ‘walking across the stage to receive my degree certificate’.

#### Goal expectancy task

In this computerised task, participants provided ratings for their nine most important goals on six goal attributes. These were as follows: how likely they were to achieve the goal in their future (likelihood); how much control they perceived themselves to have over achieving the goal (controllability); how important achieving the goal would be to their life story (importance); how vividly they could picture achieving the goal (vividness); how happy achieving the goal would make them feel (anticipated happiness); and how disappointed they would feel if they failed to achieve the goal (anticipated disappointment). All six variables were assessed on a 7-point Likert scale (0 = ‘Not at all’ to 6 = ‘Extremely’).

#### Jigsaw task

Participants chose one of 12 famous world landmark scenes (e.g., the Eiffel Tower, Niagara Falls) on an interactive jigsaw app for iPad (Sparkle Apps, 2014). Each jigsaw comprised 252 pieces. Participants had to move the pieces into place with their finger and were given 10 min to complete as much of the jigsaw as possible.

#### Visual analogue mood scales (VAMS)

Positive and negative affect were measured using printed visual analogue scales, where participants had to mark on a 100 mm line how they felt in that moment (Nelis et al., 2015). The difference between positive and negative affect was clarified by the researcher (i.e., high to low positive affect ranges from feeling joyful and energetic through to flat, tired or unmotivated, whilst high to low negative affect ranges from feeling distressed, nervous or tense through to feeling calm and relaxed). The positive scale ranged from ‘Totally not in a positive mood’ (0) to ‘Very positive mood’ (100); the negative scale ranged from ‘Totally not dejected, “down”, sad, depressed’ (0) to ‘Very dejected, “down”, sad, depressed’ (100).

#### Related positive simulation task (Related-PST)

**Fig. 1:**
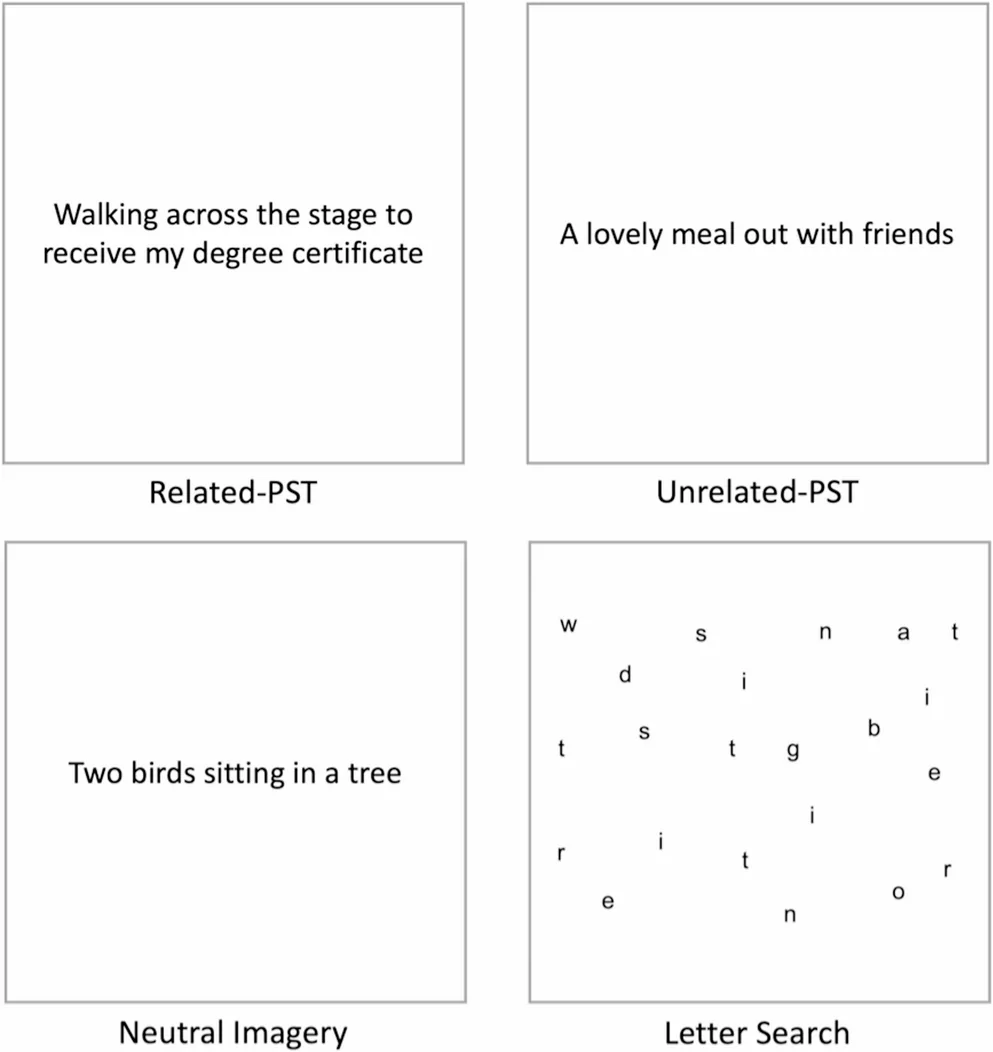
Example trials from the two PST, neutral imagery and letter search control tasks

This computerised task required participants to simulate achievement of their nine shortlisted goals from the goal production task. On each trial, an achievement event appeared onscreen for 15 s and participants were instructed that they needed to vividly imagine the event throughout this time but need not give any overt responses. Participants were also informed that some items might appear more than once. Each achievement event was repeated three times for a total of 27 trials in the main block. Prior to the main block, participants completed a practice block consisting of five generic positive simulation cues. At the end of each block, participants reported, on average, how vividly they were able to imagine the events and how emotional the events were (both on a 0–6 Likert, 0 = ‘Not at all’ to 6 = ‘Extremely’). During the main block, trials were presented in a fixed pseudorandom order. Example trials from this and the alternative experimental manipulation tasks are shown in Fig. 1.

#### Unrelated positive simulation task (Unrelated-PST)

This computerised task was identical to the related-PST task, except that instead of simulating goal achievement events, it required participants to simulate a series of generic positive future events as vividly as possible in response to cues provided onscreen. These cues were experimenter-devised and did not directly correspond to participants’ personal goals (see example in Fig. 1).

#### Neutral imagery task

This computerised task was identical to the two PST tasks except that, in this case, participants were required to visualise a series of emotionally neutral scenes as vividly as possible in response to on screen cue phrases (see Fig. 1 for example). Cue phrases were taken from Nolen-Hoeksema and Morrow (1993).

#### Letter search control task

This computerised task required participants to view a series of letter arrays containing 19–36 apparently random lower-case letters and keep count of how many arrays contained a rotated, ‘deviant’ letter. Each array in fact comprised the characters of one of the neutral imagery task cue phrases, scattered in an arbitrary pattern across the screen (see Fig. 1). The five practice trials consisted of letters from one of the unrelated-PST simulation cues scattered in the same manner. In 10 out of the 27 main block arrays (and 2 out of 5 practice arrays), one letter had been rotated by either 5, 10 or 15 degrees from the vertical. In effect, therefore, participants performed a visual search on each trial, determining if a deviant letter was present, and maintained an ongoing mental tally of the total number of deviants identified. At the end of each block, participants rated the task on emotionality (0–6 Likert as in the other three tasks) and reported the number of deviant letters they had counted.

### Procedure

**Fig. 2:**
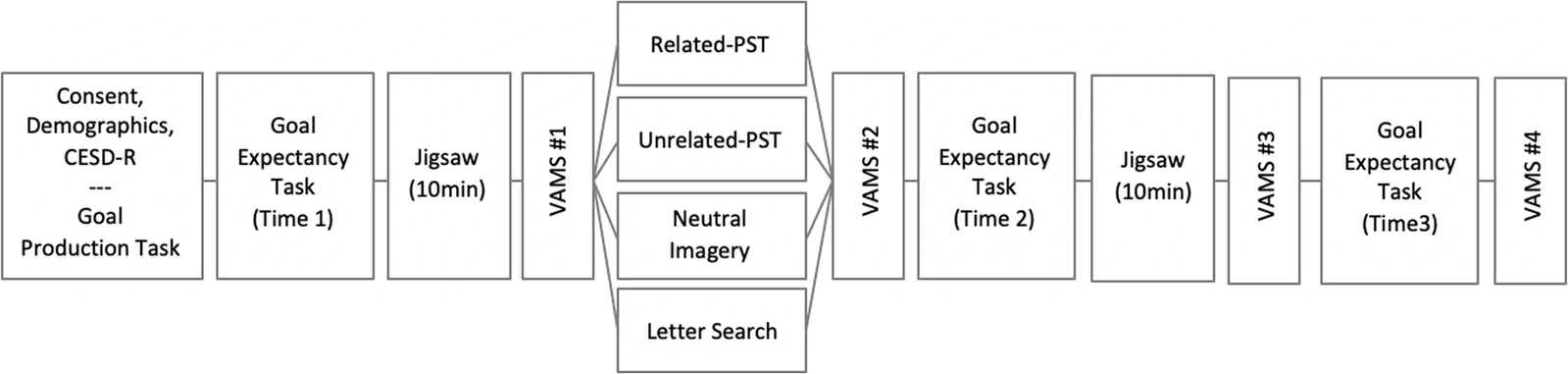
Outline of experimental procedure

Participants completed all tasks individually. The researcher provided instructions prior to each task and remained present throughout. After providing informed consent, participants first completed the CESD-R and goal production task on paper. Then, moving to the MacBook, they completed the first goal expectancy task (Time 1). They then completed the jigsaw task for 10 min, followed by the first VAMS on paper. Returning to the MacBook, participants completed their assigned PST / neutral imagery / letter search control task, followed by a second paper VAMS. They then completed the second goal expectancy task (Time 2) and further 10 min of the jigsaw task, followed by the third VAMS. Finally, they completed the last goal expectancy task (Time 3) and the fourth and final VAMS. All computerised tasks were administered using Qualtrics XM (Qualtrics, Provo, UT). The entire procedure, which lasted approximately 90 min, is outlined in Fig. 2.

### Design & analysis plan

We assessed post-intervention values of six goal ratings as dependent variables (DVs; likelihood, controllability, importance, vividness, happiness and disappointment) across four experimental groups (related-PST, unrelated-PST, neutral imagery, letter search control) and according to individual depression severity (CESD-R). Two sets of analyses were conducted, using multilevel linear models computed with the R package *lme4* (Bates et al., 2015). The first set predicted DV values at Time 2, i.e., immediately following the simulation, neutral imagery or control task, with Time 1 (baseline) values as an item-level covariate. The second set predicted DV values at Time 3, again with baseline values as a covariate, to establish whether any effects were maintained after a short delay. CESD-R scores were grand mean-centred across the four groups and used as a participant-level covariate, examining its independent effect and interactions with experimental group.

Because the tasks used in each experimental group were qualitatively different, we used dummy coding to isolate potential effects of each of the three imagery conditions (related-PST, unrelated-PST, neutral imagery, = 1) versus the letter search control (= 0). VAMS mood ratings and post-task vividness / emotionality ratings (and deviant letter counts) were examined to contextualise findings from the main multilevel analyses.

## Results

### Baseline goal ratings

Participant-level baseline scores on each of the six goal ratings (likelihood, controllability, importance, vividness, anticipated happiness, anticipated disappointment) are presented according to condition in Table 2.

**Table 2:** Baseline (Time 1) Mean Scores (SD) on goal ratings

DV	Condition				
	Related-PST	Unrelated-PST	Neutral Imagery	Letter Search	F (*p*)
Likelihood	4.67 (0.82)	4.74 (0.69)	4.95 (0.60)	4.62 (0.55)	2.20 (0.09)
Controllability	4.63 (0.76)	4.75 (0.69)	4.75 (0.61)	4.81 (0.66)	0.59 (0.63)
Importance	5.11 (0.59)	5.05 (0.59)	5.16 (0.56)	5.05 (0.59)	0.36 (0.78)
Vividness	4.69 (0.89)	4.50 (1.03)	4.78 (0.84)	4.65 (0.70)	0.84 (0.48)
Anticipated Happiness	5.41 (0.47)	5.45 (0.42)	5.32 (0.62)	5.19 (0.60)	2.18 (0.09)
Anticipated Disappointment	4.09 (1.43)	4.27 (1.00)	4.29 (1.05)	4.21 (0.86)	0.32 (0.81)

Note. Equal variances not assumed for happiness DV, *df* = 3, 100.47, and disappointment, *df* = 3, 100.41; otherwise *df* = 3, 183

*F*-values from one-way ANOVA (see Table 2) indicate that participants’ average scores were equivalent across the four conditions at Time 1. Pearson’s correlations with participant CESD-R score were computed for each rating, with none reaching significance (|*r*| < 0.14, *p* > .07).

### Experimental task ratings (vividness, emotionality, deviants)

After the experimental manipulation participants in all four conditions rated the emotionality of that task. For the three imagery-based tasks (related-PST, unrelated-PST, neutral imagery), participants also rated the vividness of the imagery produced within the task. Analysis of these ratings across conditions allowed us to evaluate alternative explanations and contextualise the results. A one-way ANOVA on vividness ratings between the three imagery conditions showed no significant effect of condition (*F*_(2, 138)_ = 2.54, *p* = .083). However, a one-way ANOVA on emotionality ratings, between all four conditions, revealed a significant effect (*F*_(3, 183)_ = 61.1, *p* < .001), with ratings dropping successively between conditions from the related-PST (M = 4.41, SD = 1.20) down to the letter search (M = 0.94, SD = 1.16). Post-hoc comparisons showed significant differences between every pair of conditions (corrected *p*s < 0.001) apart from related-PST and unrelated-PST (*t*(91) = 2.24, corrected *p* = .106). This variable was therefore included as a participant-level covariate in subsequent multilevel models.

At the end of the letter search task participants reported the number of deviants counted within the task. Scores ranged between 4 and 17, tending to slightly underestimate the true answer of 10 (*M* = 8.76, *SD* = 2.38, *Med* = 9.00). This is reported for descriptive purposes only and was not further analysed as it could not be compared across groups.

### Post-intervention differences in goal ratings

Multilevel analyses assessing goal ratings at subsequent time points were performed using REML estimation, with random intercepts by participant and an overall fixed intercept term. The three condition dummy variables were entered as fixed between-subjects factors and mean-centred CESD-R scores as a continuous participant-level predictor. Three interactions were also included in each model: Related-PST × CESD-R, Unrelated-PST × CESD-R, and Neutral-Imagery × CESD-R. This enabled us to determine whether any differences across the four conditions were dependent on depressive symptom level.

#### Immediate effects of PST/neutral imagery/control tasks

The first set of analyses, predicting DV scores at Time 2, are summarised in Table 3. All analyses included 1683 item-level observations (goals) nested within 187 participants. We take each goal rating DV in turn.

##### Likelihood

Time 2 likelihood ratings were not influenced by condition, while controlling for Time 1 baseline values (|*b*| ≤ 0.10, *p* ≥ .40). However, Time 1 ratings were a significant positive predictor of Time 2 ratings (*b* = 0.60, *p* < .001); as was task emotionality (at participant level; *b* = 0.06, *p* = .013). No other effects were significant (*p*s > 0.15).

##### Controllability

**Fig. 3:**
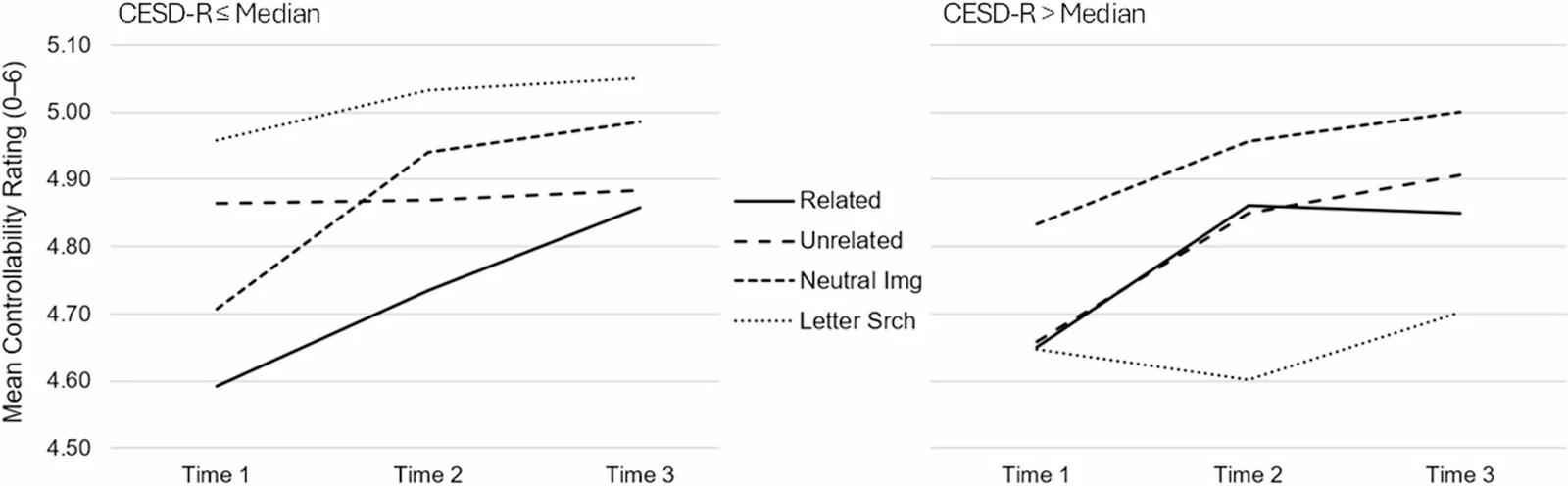
Mean controllability ratings by CESD-R Score (Low/High), condition and time. *Note.* Left panel: averages for participants scoring up to grand median (CESD-*R* ≤ 10); right panel: averages for participants scoring above grand median (CESD-*R* > 10)

Time 2 controllability ratings were not influenced by condition alone (|*b*| ≤ 0.13, *p* ≥ .14). However, CESD-R score was a significant negative predictor (*b* = -0.02, *p* < .001); while the Related × CESD-R (*b* = 0.02, *p* = .012) and Unrelated × CESD-R (*b* = 0.02, *p* < .001) interactions were also significant. Therefore, more depressed participants generally felt a lower level of control over their goals at Time 2 but experienced relatively higher control if exposed to related or unrelated PST (see Fig. 3; sharp T1–T2 increases on right panel). Additionally, Time 1 ratings were a significant positive predictor of Time 2 ratings (*b* = 0.60, *p* < .001). No other effects were significant (*p*s > 0.10).

##### Importance

Time 2 importance ratings were only significantly related to Time 1 ratings (*b* = 0.63, *p* < .001); no other effects were significant (*p*s > 0.25).

**Table 3:** Fixed effect estimates from multilevel analyses of immediate effects (Time 2)

Model DV	-2LL	Fixed Effect	Estimate (b)	Significance (*p*)
*Likelihood*	3573.3	Related Unrelated Neutral Img CESD-R Related × CESD-R Unrelated × CESD-R Neutral Img × CESD-R **Time 1 Likelihood** **Emotionality**	-0.10 -0.06 0.00 -0.01 0.00 0.01 0.00 **0.60** **0.06**	0.40 0.56 0.99 0.16 0.79 0.35 0.84 **< 0.001** **0.013**
*Controllability*	4067.8	Related Unrelated Neutral Img **CESD-R** **Related × CESD-R** **Unrelated × CESD-R** Neutral Img × CESD-R **Time 1 Controllability** Emotionality	0.10 0.06 0.13 **-0.02** **0.02** **0.02** 0.01 **0.59** -0.01	0.38 0.58 0.14 **< 0.001** **0.012** **< 0.001** 0.12 **< 0.001** 0.72
*Importance*	4064.3	Related Unrelated Neutral Img CESD-R Related × CESD-R Unrelated × CESD-R Neutral Img × CESD-R **Time 1 Importance** Emotionality	-0.05 -0.01 -0.01 -0.00 -0.00 0.00 -0.00 **0.63** 0.03	0.68 0.95 0.87 0.28 0.90 0.54 0.91 **< 0.001** 0.23
*Vividness*	4351.4	Related Unrelated Neutral Img CESD-R Related × CESD-R Unrelated × CESD-R Neutral Img × CESD-R **Time 1 Vividness** **Emotionality**	-0.17 -0.26 0.11 -0.01 0.01 0.01 0.01 **0.46** **0.12**	0.33 0.11 0.41 0.21 0.60 0.15 0.24 **< 0.001** **< 0.001**
*Anticipated Happiness*	3546.9	Related Unrelated Neutral Img **CESD-R** Related × CESD-R **Unrelated × CESD-R** **Neutral Img × CESD-R** **Time 1 Happiness** Emotionality	-0.01 0.08 0.10 **-0.01** 0.01 **0.01** **0.02** **0.59** 0.03	0.94 0.41 0.25 **0.012** 0.19 **0.039** **0.013** **< 0.001** 0.16
*Anticipated Disappointment*	5137.5	Related Unrelated Neutral Img CESD-R Related × CESD-R Unrelated × CESD-R Neutral Img × CESD-R **Time 1 Disappointment** Emotionality	-0.17 -0.05 0.24 -0.00 0.00 0.00 0.01 **0.60** 0.07	0.41 0.79 0.16 0.97 0.73 0.75 0.56 **< 0.001** 0.56

Note. Significant estimates in bold; *CESD-R* = grand mean-centred CESD-R score

##### Vividness

Time 2 vividness ratings were significantly related to Time 1 ratings (*b* = 0.46, *p* < .001) and task emotionality (*b* = 0.12, *p* < .001); no other effects were significant (*p*s > 0.10).

##### Anticipated happiness

**Fig. 4:**
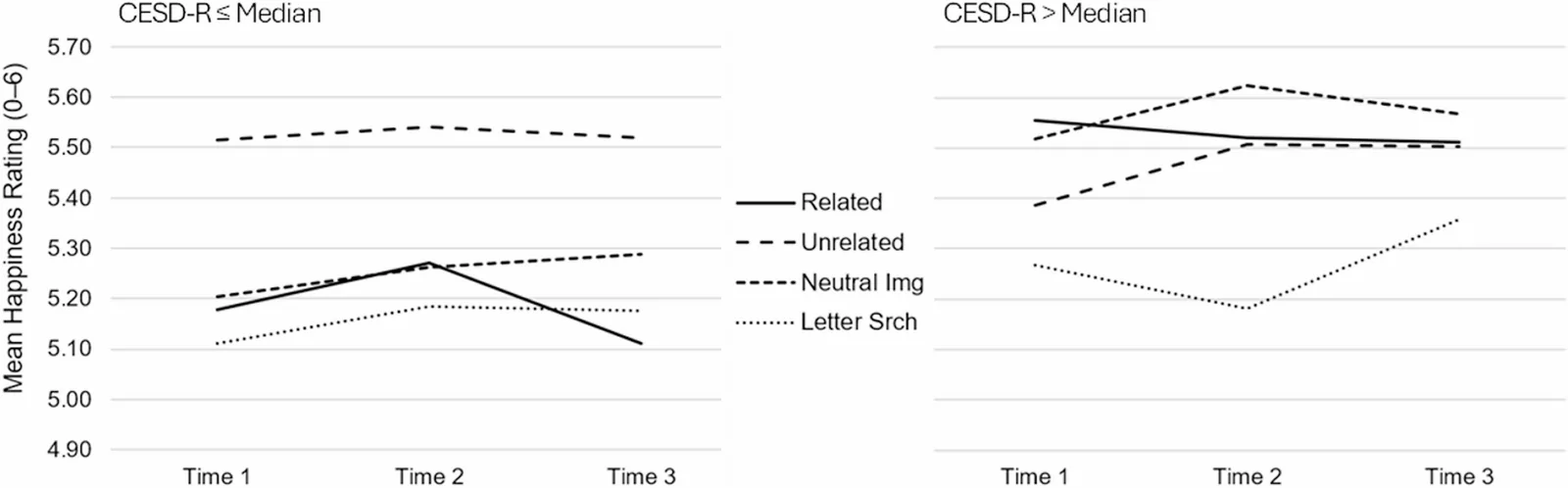
Mean anticipated happiness ratings by CESD-R Score (Low/High), condition and time. *Note.* Left panel: averages for participants scoring up to grand median (CESD-*R* ≤ 10); right panel: averages for participants scoring above grand median (CESD-*R* > 10)

Time 2 happiness ratings were not influenced by condition alone (|*b*| ≤ 0.10, *p* ≥ .25). However, CESD-R score was a significant negative predictor (*b* = -0.01, *p* = .012); while the Unrelated × CESD-R (*b* = 0.01, *p* = .039) and Neutral Imagery × CESD-R (*b* = 0.02, *p* = .013) interactions were also significant. More depressed participants generally anticipated lower levels of happiness on achieving their goals but anticipated relatively higher levels if exposed to the unrelated PST or neutral imagery interventions (see Fig. 4; T1–T2 increases in dashed lines on right panel). Additionally, Time 1 ratings were a significant positive predictor of Time 2 ratings (*b* = 0.59, *p* < .001). No other effects were significant (*p*s > 0.15).

##### Anticipated disappointment

Time 2 disappointment ratings were only significantly related to Time 1 ratings (*b* = 0.60, *p* < .001); no other effects were significant (*p*s > 0.15).

#### Maintenance of effects over a short delay

The second set of analyses, predicting DV scores at Time 3, are summarised in Table 4. Analyses reflect the same 1683 items (goals) nested within 187 participants.

##### Likelihood

Time 3 likelihood ratings were lower in the related-PST group relative to the control (*b* = -0.31, *p* = .013). As before, Time 1 ratings were a significant positive predictor at Time 3 (*b* = 0.55, *p* < .001); as was task emotionality (*b* = 0.08, *p* < .001). No other effects were significant (*p*s > 0.10).

##### Controllability

Time 3 controllability ratings were not influenced by condition alone (|*b*| ≤ 0.06, *p* ≥ .56). However, CESD-R score was a significant negative predictor (*b* = -0.01, *p* = .015); while the Unrelated × CESD-R (*b* = 0.02, *p* = .007) interaction was also significant. Therefore, more depressed participants continued to feel a lower level of control over their goals but experienced relatively higher control if exposed to unrelated PST (see Fig. 3). Additionally, Time 1 ratings were a significant positive predictor of Time 2 ratings (*b* = 0.51, *p* < .001). No other effects were significant (*p*s > 0.15).

**Table 4:** Fixed effect estimates from multilevel analyses of delayed effects (Time 3)

Model DV	-2LL	Fixed Effect	Estimate (b)	Significance (*p*)
*Likelihood*	3541.7	**Related** Unrelated Neutral Img CESD-R Related × CESD-R Unrelated × CESD-R Neutral Img × CESD-R **Time 1 Likelihood** **Emotionality**	**-0.31** -0.18 -0.18 -0.01 -0.00 0.01 0.01 **0.55** **0.08**	**0.013** 0.11 0.85 0.11 0.98 0.14 0.46 **< 0.001** **< 0.001**
*Controllability*	3966.4	Related Unrelated Neutral Img **CESD-R** Related × CESD-R **Unrelated × CESD-R** Neutral Img × CESD-R **Time 1 Controllability** Emotionality	-0.04 -0.06 0.06 **-0.01** 0.01 **0.02** 0.01 **0.51** 0.02	0.78 0.63 0.56 **0.015** 0.20 **0.007** 0.35 **< 0.001** 0.35
*Importance*	3898.4	Related Unrelated Neutral Img CESD-R Related × CESD-R Unrelated × CESD-R Neutral Img × CESD-R **Time 1 Importance** **Emotionality**	-0.22 -0.12 -0.07 -0.01 0.00 0.00 0.00 **0.60** **0.05**	0.05 0.28 0.47 0.20 0.86 0.23 0.75 **< 0.001** **0.021**
*Vividness*	4132.2	**Related** **Unrelated** Neutral Img CESD-R Related × CESD-R Unrelated × CESD-R Neutral Img × CESD-R **Time 1 Vividness** **Emotionality**	**-0.57** **-0.42** -0.17 0.00 -0.01 0.01 0.01 **0.43** **0.16**	**0.005** **0.025** 0.92 0.98 0.53 0.60 0.55 **< 0.001** **< 0.001**
*Anticipated Happiness*	3523.1	**Related** Unrelated Neutral Img CESD-R Related × CESD-R Unrelated × CESD-R Neutral Img × CESD-R **Time 1 Happiness** **Emotionality**	**-0.34** -0.15 -0.06 -0.00 0.00 0.00 0.01 **0.57** **0.09**	**0.003** 0.15 0.49 0.55 0.94 0.72 0.13 **< 0.001** **< 0.001**
*Anticipated Disappointment*	4879.8	**Related** Unrelated Neutral Img CESD-R Related × CESD-R Unrelated × CESD-R Neutral Img × CESD-R **Time 1 Disappointment** Emotionality	**-0.46** -0.33 -0.11 0.00 0.01 0.01 0.01 **0.55** 0.07	**0.026** 0.09 0.52 0.75 0.62 0.62 0.57 **< 0.001** 0.07

Note. Significant estimates in bold; *CESD-R* = grand mean-centred CESD-R score

##### Importance

Time 3 importance ratings were only significantly related to Time 1 ratings (*b* = 0.60, *p* < .001) and task emotionality (*b* = 0.05, *p* = .021); no other effects were significant (*p*s > 0.05).

##### Vividness

Time 3 vividness ratings were lower in the related-PST (*b* = -0.57, *p* = .005) and unrelated-PST groups relative to the control (*b* = -0.42, *p* = .025). Time 1 ratings were a significant positive predictor at Time 3 (*b* = 0.43, *p* < .001); as was task emotionality (*b* = 0.16, *p* < .001). No other effects were significant (*p*s > 0.50).

##### Anticipated happiness

Time 3 happiness ratings were lower in the related-PST group relative to the control (*b* = -0.34, *p* = .003). As before, Time 1 ratings were a significant positive predictor (*b* = 0.57, *p* < .001); as was task emotionality (*b* = 0.09, *p* < .001). No other effects were significant (*p*s > 0.10).

##### Anticipated disappointment

Time 3 disappointment ratings were lower in the related-PST group relative to the control (*b* = -0.46, *p* = .026). Time 1 ratings were a significant positive predictor (*b* = 0.55, *p* < .001); no other effects were significant (*p*s > 0.05).

### Momentary mood across the experiment

Finally, we examined participants’ VAMS mood reports throughout the experiment: VAMS-1 (immediately before intervention), through VAMS-2 (immediately after) and VAMS-3 (after second goal expectancy task), to VAMS-4 (end of experiment). If mood changed differentially between conditions, this might explain findings from the multilevel analyses of goal ratings. Firstly, then, we computed a 4 (Time Point) × 4 (Condition) mixed ANCOVA, with repeated measures on the time point factor and CESD-R score as a covariate, on *positive* mood scores. This produced a significant effect of time point (*F*_(3, 534)_ = 10.78, *p* < .001, η^2^_p_ = 0.06), with positive mood increasing from VAMS-1 (*M* = 72.1, *SD* = 21.2) to VAMS-4 (*M* = 80.0, *SD* = 19.4). The main effect of condition was non-significant (*F*_(3, 178)_ = 2.16, *p* = .094), although the effect of the covariate, CESD-R, was significant (*F*_(1, 178)_ = 6.64, *p* = .011, η^2^_p_ = 0.04). CESD-R was negatively correlated with positive mood at the end (VAMS-4; *r* = − .20, *p* = .006) but not the start of the experiment (VAMS-1; *r* = − .04, *p* = .59). Neither interaction, Time Point × Condition or Time Point × CESD-R, was significant (*F*s < 2, *p*s > 0.18).

We then computed a similar 4 (Time Point) × 4 (Condition) mixed ANCOVA, with CESD-R score as covariate, on *negative* mood scores. This again produced a significant effect of time point (*F*_(3, 537)_ = 4.28, *p* = .005, η^2^_p_ = 0.02), with negative mood decreasing from VAMS-1 (*M* = 18.5, *SD* = 20.2) to VAMS-4 (*M* = 14.2, *SD* = 17.7). The main effect of condition was non-significant (*F* < 1), although the effect of the covariate, CESD-R, was significant (*F*_(1, 179)_ = 19.76, *p* < .001, η^2^_p_ = 0.10). CESD-R was positively correlated with negative mood at the start (VAMS-1; *r* = .22, *p* = .002) and end of the experiment (VAMS-4; *r* = .31, *p* < .001). Neither interaction, Time Point × Condition or Time Point × CESD-R, was significant (*F*s < 1). These analyses suggest that there were no meaningful group differences in momentary mood according to experimental group at any point in the experiment; although positive mood tended to increase, and negative mood decrease, across groups. Negative mood ratings were consistently related to depression scores.

## Discussion

The current investigation explored the impact of brief positive simulation training (PST) on cognitive and emotional expectancies related to personal goals. Specifically, it compared post-manipulation goal-related expectancies, accounting for baseline pre-manipulation expectancies, across four experimental conditions: goal-related PST; goal-unrelated PST; neutral imagery task; or a letter search control task. The inclusion of the neutral imagery and letter search control conditions allowed us to test whether it is positive episodic simulation, rather than generic imagery engagement, that affects goal-related expectancies. Our primary hypothesis was that participants who undertook brief inductions in PST, either goal-related or goal-unrelated, would report significantly higher post-manipulation goal expectancies compared to those who completed the neutral imagery and letter search control tasks. Furthermore, we argued that if improvements were evident only after the goal-related PST task then this would lend support to the argument that positive episodic simulation serves as a “motivational amplifier”. In contrast, if comparable effects were observed following both PST tasks, this would support the view that effects arise because positive episodic simulation fosters a broader optimistic orientation. We also investigated whether any effects of PST were sustained after a short delay, and whether any effects of PST differed as a function of depressive symptomatology.

We found that expectancy ratings provided immediately post-manipulation (T2) were not significantly higher, after accounting for baseline pre-manipulation expectancy ratings, in any of the four experimental manipulations. However, there were significant interactions between experimental condition and CESD-R score that lend partial support to our hypotheses. PST, both goal-related and unrelated, lead to higher ratings of perceived controllability for those experiencing high levels of depressive symptoms. This effect was sustained after a short delay (T3) for participants in the unrelated-PST condition. Furthermore, unrelated-PST and the neutral imagery task led to higher ratings of anticipated happiness immediately post-manipulation; again, these effects were evident in those experiencing higher levels of current depressive symptoms. However, neither of these effects were sustained after a short delay. Surprisingly, for several dependent variables, the expectancy ratings provided after a short delay post-manipulation (T3) were significantly lower, after accounting for baseline pre-manipulation expectancy ratings, in the related-PST condition. This effect emerged for ratings of likelihood, vividness, anticipated happiness and anticipated disappointment. Thus, participants in this condition rated their goal achievement as less vivid and likely to happen, that achievement would bring less happiness, and that failure would be accompanied by lower levels of disappointment. These findings are counter to our hypotheses where we expected engaging in related-PST to lead to improvements in expectancy ratings.

The key variable that evidenced support for the primary hypothesis was controllability. Previous research suggests that high controllability is a key attribute of meaningful goals, without which motivation is likely to be disrupted (Clayton McClure & Cole, 2022). Previous literature has demonstrated that training in episodic simulation can make future events feel more controllable (Boland et al., 2018; Hallford et al., 2022b). Our findings supported and extended this previous literature, demonstrating that episodic simulation can improve perceived controllability of participants’ personal goals.

The findings that both related and unrelated PST led to positive changes in perceived controllability of goal achievement is consistent with previous work suggesting that the effects of positive episodic simulation can generalise to expectancies about future events beyond those that form part of the simulation training itself (Anderson et al., 2024; Boland et al., 2018; Hallford et al., 2023). The potential for positive episodic simulation to modify an individual’s general optimistic orientation is useful from a therapeutic context because it suggests that interventions incorporating episodic simulation do not necessarily need to be wholly personalised. Rather, more general training in how to incorporate more episodic imagery when thinking about the future might be sufficient.

One caveat of note is that the goal-unrelated PST was not individualised for each participant; instead, it was a generic set of positive cue phrases that were unrelated to personal goals when compared to the simulation cues used within the goal-related PST condition, where cues had been generated by participants to represent achievement of their personal goals. Therefore, the goal-unrelated PST may have been relevant to some participants’ personal goals and the extent to which this was the case would have varied across participants. However, this does not detract from the argument that our findings support the validity of general training in positive episodic simulation as a potential therapeutic tool. With this in mind, it is interesting to note that the only sustained benefit, evidenced by higher ratings at T3, was for controllability within the goal-unrelated PST condition. We are mindful of overstating the significance of this finding, but it does lend support to the argument that general training in positive episodic simulation shows promising benefit.

The finding that PST seemed to confer particular benefit for those experiencing higher levels of depressive symptomology is pertinent given that depression is associated with difficulties with positive episodic simulation (e.g., Bjärehed et al., 2010; Holmes et al., 2008; MacLeod et al., 1997; MacLeod & Byrne, 1996; MacLeod & Salaminiou, 2001; Morina et al., 2011; Stöber, 2000) and that these difficulties arguably sit at the heart of a pessimistic predictive style that feeds into goal-related motivational difficulties (Anderson et al., 2023; Dickson et al., 2011; Gamble et al., 2021). It was, indeed, the case that those experiencing higher levels of depression reported lower levels of controllability at T2 and T3, but that that these perceptions of uncontrollability about goal achievement were mitigated for those who had completed either of the positive episodic simulation tasks. As such, our findings add further weight to the argument that training in episodic simulation may be a useful tool in changing the pessimistic outlook evident in individuals experiencing elevated levels of depression.

With respect to the other goal ratings, the findings were more mixed. Previous literature has suggested that episodic simulation can enhance anticipated pleasure (Hallford et al., 2022b; Ji et al., 2021; Renner et al., 2019). We, therefore, predicted that a similar effect would emerge within our study. Whilst we found that unrelated-PST led to higher ratings of anticipated happiness immediately post-manipulation, this was also true of the neutral imagery task. Again, these effects were only evident in those experiencing higher levels of depressive symptoms. However, neither of these effects were sustained after a short delay. The finding that engaging in a visual imagery-based task, rather than episodic simulation specifically, affected ratings of anticipated happiness for personal goals is somewhat difficult to interpret in the context of previous literature. While research has demonstrated the effects of episodic simulation relative to non-imagery control tasks, often verbal or mathematical in nature (e.g. Hallford et al., 2022a; Ji et al., 2021; Renner et al., 2019), two previous studies have demonstrated effects relative to a very similar neutral visual imagery task (Anderson et al., 2024; Boland et al., 2018). Analyses of mood ratings across the study suggest that changes in anticipated happiness cannot be explained by condition-dependent changes in momentary positive/negative affect. One possibility is that the neutral visual imagery task unintentionally led participants to engage in episodic simulation that was positive in nature; the task requires scene construction (e.g. layout of local shopping centre) so this may have elicited episodic simulation processes. Conversely, the letter search control task may have served to block mental imagery processes by taxing visuospatial working memory (Baddeley & Andrade, 2000). However, if engaging in neutral visual imagery is sufficient to elicit episodic simulation processes then this raises questions as to why it only affected anticipated happiness ratings and why it had such an effect in this, but not earlier, studies.

Analyses exploring ratings of likelihood, importance, vividness and anticipated disappointment did not lend any support to our hypotheses; no significant benefits of PST were found at T2. The finding regarding anticipated disappointment is novel with respect to the effects of episodic simulation training on anticipated negative emotions. However, the other findings conflict with previous studies that have found that positive episodic simulation makes positive future events more vivid in one’s mind, as well as feeling more important and likely to occur (e.g., Boland et al., 2018; Hallford, Farrell et al., 2022).

One potential explanation for inconsistencies with previous findings is that the simulation duration used within the current study lasted only 15s per event. This is shorter than durations used within some other experimental work (e.g., Hallford, Farrell et al., 2022) and, arguably, this duration may not be sufficient for participants to engage in the vivid and detailed pre-experiencing necessary to influence expectancies about future events. However, Boland et al. (2018) used an almost identical procedure to that within the current study and they found beneficial changes in expectancies about generic future events. Therefore, whilst a longer duration should be investigated within future work, we cannot unequivocally suggest this as the only potential reason why our findings are inconsistent with previous studies examining the effects of episodic simulation on expectancies of future events.

An alternative explanation for the inconsistencies with previous findings is that this is the first study to specifically focus on expectancies about personal goals. It is possible, that personal goals are inherently different to the more generic future events used in previous research. Theoretically, it has been argued that autobiographical thinking is underpinned by a remembering-imagining system that integrates past, current and future-goal related activities (Conway, 2009; Conway et al., 2016). Within this system, the salience of past and future events is closely tied to current goals. As such, it is likely that personal goals were less novel stimuli around which to generate expectancies; many of the goals generated within the current study may have been developed and even mentally simulated prior to participation. Being mental representations of aims, dreams and ambitions, personal goals are very likely important and pleasurable to the holder of those goals. Moreover, the current study focused specifically on those goals selected as ‘most important’ across three future time periods. Whilst baseline ratings of likelihood of achievement for personal goals were not as high as those for importance and anticipated happiness, they were higher than those seen in other studies using comparable ratings scales with generic positive future events (e.g., Boland et al., 2018). Therefore, our participants may have selected goals that they were relatively confident could be achieved.

A secondary aim of our study was to investigate whether any effects of PST on goal ratings were sustained after a delay of ten minutes. With respect to perceived controllability the effects we observed were not wholly transitory and did last for this ten-minute period post-simulation for participants who undertook goal-unrelated PST. From a therapeutic perspective this is important because, although regular practice is likely to be a feature of any intervention, the effects of PST would need to be sustained between practice sessions. Several studies have demonstrated that Future Specificity Training, a structured intervention that encourages episodic simulation processes, has a sustained impact on expectancies about the future, including increased (Bogaert et al., 2024; Hallford et al., 2020, 2023). Our findings add to this literature by suggesting that the effects of episodic simulation on expectancies about personal goal achievement may also be sustained over time. However, it is important to note that our study only investigated the relatively short-term endurance of these effects following a single experimental induction. Thus, further work needs to fully delineate the duration of the effects of imagery-based interventions on personal goals.

An unexpected finding arose at T3 regarding the effects of engaging in goal-related simulations. Participants who completed the goal-related PST, relative to those in the letter search control task, rated their goal achievement as less vivid and likely to happen, that achievement would bring less happiness, and that failure would be accompanied by lower levels of disappointment. This was contrary to our hypotheses where we expected engaging in related-PST to lead to improvements in expectancy ratings. Our findings could be explained by research suggesting that mental simulation can act as a substitute for experience and, in doing so, reduces motivation for goal achievement (Kappes & Morewedge, 2016). This work has found that mentally simulating success can make individuals less likely to succeed compared to those who simulate failure or encountering problems on the path to goal achievement (Kappes et al., 2012; Kappes & Oettingen, 2011). Furthermore, other work has suggested that process-based, rather than outcome-based, mental simulations may be more effective in eliciting goal-oriented motivation and behaviours (Pham & Taylor, 1999). Therefore, goal-focused interventions may need to think carefully about how they incorporate episodic simulation, focusing on the incorporation of process-based simulations and the simulation of overcoming obstacles, rather than merely visualising success.

Across the course of the experiment, participants provided a series of ratings of momentary mood. In addition, after completing the experimental manipulation, all participants were asked to rate the task’s emotionality. Furthermore, those who completed one of the three imagery-based tasks were asked to rate the vividness of their mental simulations. These ratings allowed us to evaluate alternative explanations and contextualise the results. Analyses found that negative mood decreased and positive mood increased across the course of the experiment; however, these changes were independent of the experimental task manipulation. Furthermore, the mental simulations were rated equivalently for vividness across the three imagery-based tasks. Therefore, neither seem likely explanations for our current findings. However, the post-manipulation emotionality ratings did differ between experimental conditions. The related-PST and unrelated-PST were judged to be elicit more emotions than the neutral imagery and letter control tasks. In light of this, we did include participant-level emotionality as a covariate within analyses to account for its potential effect on any significant findings.

Analyses suggested that task emotionality did not significantly impact the perceived controllability of personal goals; therefore, it does not explain the beneficial effects of positive episodic simulation with regards this expectancy. However, task emotionality, did affect ratings of vividness and likelihood. Participants who rated the experimental manipulation as more emotional provided post-manipulation expectancies whereby goal achievement was more vivid and likely. This raises questions about the importance of individual differences and engagement in episodic simulation tasks. Some individuals may have a natural tendency to produce more emotional mental simulations than others and, potentially, that these individuals might derive more benefit. Evidence suggests that individual differences exist in other aspects of mental imagery, such as the frequency and vividness of imagery generation and the extent to which individuals spontaneously use images in their everyday lives (e.g., Floridou et al., 2022), Furthermore, a small percentage of the population report that they are incapable of voluntary generating mental images, termed aphantasia (Zeman et al., 2015). It is feasible that these individual differences could impact the potential usefulness of episodic simulation as a therapeutic tool, particularly among those who find mental imagery formation particularly difficult or even impossible. We did not measure any of these individual differences in imagery ability, or screen for aphantasia, within the current study. This is a limitation of the current research and presents interesting questions to be investigated within future work.

It is important to acknowledge several other limitations to the current study and resultant future research directions. The current study did not include a non-imagery goal-oriented condition, e.g. goal-related semantic elaboration. Therefore, whilst positive episodic simulation appeared to confer benefits compared to generic imagery and non-imagery tasks, we cannot be certain that other goal-oriented thinking might be as useful as positive episodic simulation. Furthermore, the current study only examined the cognitive and emotional expectancies relating to personal goals. We did not ask about participants’ motivation to achieve these goals, nor did we examine subsequent goal-related behaviours. Both Renner et al. (2019) and Ji et al. (2021) have shown that episodic simulation can amplify both motivation and behavioural engagement for rewarding activities. However, these studies did not explicitly examine participants’ personal goals. Therefore, future research should establish whether training in episodic simulation also leads to changes in goal-related motivation and behavioural engagement. Additional research could also explore these effects within participants who meet the criteria for major depressive disorder. It is possible that a certain level of symptomology is necessary to avoid the baseline ceiling effects on some measures that may have limited the effectiveness of our PST manipulations and, hence, our ability to gauge effectiveness of the training. A wider range of depressive symptom levels would allow us to establish whether this is the case. It is important to highlight that the current study investigated the effects of a *single induction* of episodic simulation on goal-related predictions *over relatively short intervals*. Methodologically, future research should consider the optimal approach within such induction tasks. Whilst previous research has also employed an approach akin to the current work, whereby participants completed a single block of simulation training where they repeatedly engage in a series of very brief simulations (Anderson et al., 2024; Boland et al., 2018), other research has required participants to engage in fewer, yet longer, simulations (Hallford, Farrell et al., 2022). Both have evidenced beneficial effects within experimental paradigms, yet it would be useful to consider the optimal duration of simulation that maximises vivid and detailed pre-experiencing whilst maintaining participant engagement both within and outside of the laboratory. Furthermore, another useful direction for future research would be to investigate the effects of repeated episodic simulation over much longer timeframes and/or to examine the effect of a newly developed intervention, Future Specificity Training (Bogaert et al., 2024; Hallford et al., 2020, 2023), on beliefs, motivations and behaviours pertaining to personal goals. Finally, the current study was not pre-registered, and this would also be a recommendation for any future work.

To summarise, PST affected some of the expectancies related to personal goals. Specifically, the achievement of personal goals was rated as more controllable and bringing more happiness following PST, regardless of whether PST was related or unrelated to the goals. In contrast, a neutral visual imagery task and a non-imagery control task (letter search) did not influence these expectancies. These effects were, however, only evident for participants with high levels of depressive symptoms. In contrast with previous research, other expectancies, such as likelihood and importance, were not enhanced by PST. These findings suggest that personal goals are not as amenable to change as the generic future events used in previous studies. Future research could prompt participants to generate goals that they foresee as difficult to achieve where the likelihood of such goals may provide more scope for improvement in response to training in episodic simulation.

## Data Availability

Study data and materials are openly available on the Open Science Framework DOI 10.17605/OSF.IO/J9MZ2. [https://osf.io/j9mz2/].

## Declarations

### Public significance

Future-oriented imagery techniques are a potentially useful resource that may help motivation and personal goal attainment. They are often incorporated within cognitive-behavioural therapeutic (CBT) interventions. The current study explores whether the extent to which imagery is directly related to goals is important in the success of such techniques, with findings being of potential interest to individuals, such as mental health clinicians, designing interventions to improve future-oriented thinking and behaviours.
